# Statistical Mechanics-Based Schrödinger Treatment of Gravity

**DOI:** 10.3390/e21070682

**Published:** 2019-07-12

**Authors:** Angelo Plastino, M. C. Rocca

**Affiliations:** 1 Departamento de Física, Universidad Nacional de La Plata, 1900 La Plata, Argentina; 2 Consejo Nacional de Investigaciones Científicasy Tecnológicas (IFLP-CCT-CONICET)-C. C. 727, 1900 La Plata, Argentina; 3 SThAR - EPFL, 1022 Lausanne, Switzerland; 4 Departamento de Matemática, Universidad Nacional de La Plata, 1900 La Plata, Argentina

**Keywords:** fermions, baryons, emergent entropic force, schrödringer equation, gravitation

## Abstract

The entropic gravity conception proposes that what has been traditionally interpreted as unobserved dark matter might be merely the product of quantum effects. These effects would produce a novel sort of positive energy that translates into dark matter via $E=mc^{2}$. In the case of axions, this perspective has been shown to yield quite sensible, encouraging results [DOI:10.13140/RG.2.2.17894.88641]. Therein, a simple Schrödinger mechanism was utilized, in which his celebrated equation is solved with a potential function based on the microscopic Verlinde’s entropic force advanced in [Physica A **511** (2018) 139]. In this paper, we revisit this technique with regards to fermions’ behavior (specifically, baryons).

## 1. Introduction

### 1.1. Emergent Entropy

In 2011, Verlinde [1] conceived the notion of linking gravity to an entropic force. This idea was later proved valid [2], in a classical setting.

In [1], gravity crops up as a result of information concerning the position of material bodies, joining a thermal treatment of gravitation with ’t Hooft’s holographic principle. In such terms, gravitation ought to be regarded as an emergent phenomenon. These ideas of Verlinde’s were given great attention. For example, look at [3,4,5,6,7]. An excellent review of the statistical mechanics of gravity was given by Padmanabhan [8].

Verlinde’s conceptions motivated works on cosmology, the dark energy hypothesis, cosmological acceleration, cosmological inflation, and loop quantum gravity. The corresponding literature is very ample [5,6,7]. Here, we’d like to cite Guseo’s work [9], which showed that the local entropy function, linked to a logistic distribution, turns out to be catenary and vice versa. This is an invariance that can be connected to the Verlinde’s conjecture mentioned above. Guseo advances an original interpretation of the local entropy in a system [9].

### 1.2. Our Goals in Using Schrödinger’s Equation (SE)

Verlinde depicts gravity as an emergent phenomenon that originates in the quantum entanglement between small bits of space–time information [10]. Gravitation, viewed á la Verlinde as an emergent force, deviates at very short distances from Newton’s form. The ensuing new gravitational potential, if introduced now into Schrödinger’s equation (SE), should yield quantified states, and the associated energies would constitute a novel energy source, not contemplated until now, save for our precedent axion treatment of Reference [11].

Herein, we proceed on the basis of a previous analysis [12] which uses the statistical treatment of quantum fermion gases. We applied the process described above to [12] and found a fermion–fermion gravitation force therefrom (here specifically, a baryon–baryon force). It turned out to be, as expected, proportional to $1/r^{2}$ for distances larger than one micron, but for smaller distances, novel, more involved contributions arose. Accordingly, the ensuing potential V(r) differed from the Newtonian one at short distances. We now present below the SE for *such* V(r) and solve it, expecting to find new, until now unknown quantum gravitational states for baryons.

### 1.3. Organizing Our Material

In Section 2, we review the relevant details of [12]. We suitably approximate V(r) so as to proceed in analytic fashion and set $V\left(r\right)=\sum_{i=1}^{3}V_{i}\left(r\right)$. Our central discussion is given in Section 3. Therein, we solve the ensuing Schrd̎inger’s equation for the baryon–baryon, Verlinde-like gravitation potential, separately for each of its pieces. The piece $V_{1}$ becomes protagonist and yields our most important new findings. In order to illustrate how to tackle our problem, we put forward a perhaps daring conjecture concerning dark matter in Section 4. Rough numerical estimates can be obtained. We end with some conclusions in Section 4.

## 2. Quantum Gravitational Potential $E_{P}\left(r\right)$ to Be Introduced in the SE

### 2.1. The Gravitational Potential Function for *N* Baryons of Mass *m*

It was first derived in [12], where the following four constants were introduced, for *N* baryons of mass *m*, *G* is the gravitation’s constant and $k_{B}$ Boltzmann’s constant:

- *a* and *b* in the fashion;
- $a={\left(3N\right)}^{\frac{5}{2}}h^{3}$;
- $b=32\pi {\left(\pi emK\right)}^{\frac{3}{2}}$, with a total baryons energy *K*;
- $K=10^{53}c^{2}$ Joules [13].

One ascertains in [12] that $\frac{\lambda 3Nk_{B}}{8\pi }=\frac{2}{3}Gm^{2},$ and the potential energy $E_{P}\left(r\right)$ acquires the form

(1) $$\begin{array}{c} E_{P}\left(r\right)=-Gm^{2}\frac{2b}{3a}\left\{\frac{r^{2}}{2}\ln\left(1-\frac{a}{br^{3}}\right)-\frac{a^{\frac{2}{3}}}{2b^{\frac{2}{3}}}\left\{\frac{1}{2}\ln\left[\frac{{\left[r-{\left(\frac{a}{b}\right)}^{\frac{1}{3}}\right]}^{2}}{r^{2}+{\left(\frac{a}{b}\right)}^{\frac{1}{3}}r+{\left(\frac{a}{b}\right)}^{\frac{2}{3}}}\right]\right.\right.+\\ \left.\left.\sqrt{3}\left[\arctan\left[\frac{2r+{\left(\frac{a}{b}\right)}^{\frac{1}{3}}}{\sqrt{3}{\left(\frac{a}{b}\right)}^{\frac{1}{3}}}\right]-\frac{\pi }{2}\right]\right\}\right\}, \end{array}$$

a critical result for us.

### 2.2. A Taylor Approximation (TA) for V(r)

One cannot analytically tackle the SE with such an awful $E_{P}$. Thus, we are forced to approximate V(r) piecewise in four different regions: $0<r<r_{0}$, $r_{0}<r<r_{1}$, $r_{1}<r<r_{2}$, and $r>r_{2}.$$r_{1}$ are made explicit below. $r_{0}$ is conjectured by us as being $10^{-10}$ centimeters, and $r_{2}={\left(a/b\right)}^{\frac{1}{3}}$.

(2) $$V\left(r\right)\approx V_{1}\left(r\right)+V_{2}\left(r\right)+V_{3}\left(r\right)+V_{4}\left(r\right).$$

For convenience, we define the quantity $V_{0}$

(3) $$V_{0}=-Gm^{2}{\left(\frac{b}{a}\right)}^{\frac{1}{3}}\frac{7\pi }{6\sqrt{3}},$$

and call $V_{1}$ the TA, at zeroth order, for very small *r*. *H* stands for the Heaviside function.

(4) $$V_{1}\left(r\right)=-Gm^{2}{\left(\frac{b}{a}\right)}^{\frac{1}{3}}\frac{7\pi }{6\sqrt{3}}H\left(r_{0}-r\right)=V_{0}H\left(r_{0}-r\right),$$

i.e., $V=V_{1}$ for $r<r_{0}$.

For large *r*, the pertinent approximation was obtained in [12]:

(5) $$V_{3}\left(r\right)=-\frac{Gm^{2}}{r}\left[H\left(r-r_{1}\right)-H\left(r-r_{2}\right)\right],$$

i.e., $V=V_{3}$ for $r>r_{2}$. For intermediate r−values, $r_{0}<r<r_{1}$, there is experimental evidence to choose $r_{1}=25$ micrometers [14]. We call W(r) the harmonic interpolating form between the two fixed distance values $r_{1}-r_{0}$. Thus,

(6) $$V_{2}\left(r\right)=W\left(r\right),$$

i.e., $V=V_{2}=W$ for $r_{1}<r<r_{2}$. For $V_{4}\left(r\right)$, we have

(7) $$V_{4}\left(r\right)=\frac{2Gm^{2}}{3r}H\left(r-r_{2}\right),$$

i.e., $V=V_{4}$ for $r>r_{2}$. We solve below the SE for these four potentials.

## 3. Exact Solution of the SE

We deal with the (complete) SE and call $m_{r}$ the reduced mass. One finds

(8) $$U^{\prime \prime }\left(r\right)+\left[-\frac{l(l+1)}{r^{2}}-\frac{2m_{r}}{\hbar^{2}}V\left(r\right)+\frac{2m_{r}}{\hbar^{2}}E\right]U\left(r\right)=0,$$

and analytically solves it piecewise.

### 3.1. $V_{1}$’s Exact Treatment

Let ϕ be the confluent hyper-geometric function [15]. For $V_{1}$, one has for $E>V_{0}$ and (a definition to be used below) $s=\sqrt{\frac{8m_{r}\left(E-V_{0}\right)}{\hbar^{2}}}r$:

(9) $$U_{1}^{\prime \prime }\left(r\right)+\left[-\frac{l(l+1)}{r^{2}}+\frac{2m_{r}}{\hbar^{2}}\left(E-V_{0}\right)\right]U_{1}\left(r\right)=0,$$

whose solution is that *A* and *B* are two arbitrary constants:

(10) $$U_{l1}\left(r\right)=A{\left(-is\right)}^{l+1}e^{-is}\phi \left(l+1,2l+2;-is\right)-B{\left(is\right)}^{l+1}e^{is}\phi \left(l+1,2l+2;is\right).$$

Thus, the radial solution $R_{l1}\left(r\right)$ adopts the appearance

(11) $$R_{l1}\left(r\right)=A{\left(-is\right)}^{l+1}\frac{e^{-is}}{r}\phi \left(l+1,2l+2;-is\right)-B{\left(is\right)}^{l+1}\frac{e^{is}}{r}\phi \left(l+1,2l+2;is\right),$$

and appealing to [15] and calling J to the Bessel function [15]

(12) $$\phi \left(l+1,2l+2;-is\right)=2^{2l+1}e^{-i\pi (l+\frac{1}{2})}\Gamma \left(l+\frac{3}{2}\right)s^{-\left(l+\frac{1}{2}\right)}e^{-i\frac{s}{2}}J_{l+\frac{1}{2}}\left(\frac{s}{2}\right),$$

so that

(13) $$R_{l1}\left(r\right)=2^{2l+1}\Gamma \left(l+\frac{3}{2}\right)\frac{s^{-\frac{1}{2]}}}{r}\left(Be^{\frac{3\pi il}{2}}e^{3is2}-Ae^{-\frac{3\pi il}{2}}e^{-3is2}\right)J_{l+\frac{1}{2}}\left(\frac{s}{2}\right).$$

**Boundary conditions (BC)**. $R_{l}$ must satisfy $R_{l}\left(r_{0}\right)=0$ and $R_{l}^{{}^{\prime }}\left(r_{0}\right)=0$. The two BEs now become

(14) $$J_{l+\frac{1}{2}}\left(\frac{s_{0}}{2}\right)=0,$$

requiring $s_{0}/2$ to be a zero of the Bessel function. This zero is denoted by $\chi_{l,n}$ [15].

(15) $$s_{0}=2\chi_{l,n}$$

Energy is duly quantified and reads

(16) $$E_{l,n}=\frac{\hbar^{2}}{2m_{r}}\frac{\chi_{l,n}^{2}}{r_{0}^{2}}+V_{0}.$$

This energy expression is original in the baryonic scene, having been discovered right here. We are particularly interested below in the ground state energy $E_{l=0,\;n=1}.$

Referring now to Equations (3) and (16), and taking into account that a nucleon’s mass is ∼$1.6\times 10^{-27}$ Kg, we get the $m_{r}$-value. We have as a result $E_{0,1}∼10^{-21}$ Joule. As a consequence, we obtain $V_{0}<<E_{0,1}$. Since $mc^{2}=1.44\times 10^{-10}$ Joule, we have $E_{0,1}<<mc^{2}$. For axions, the last inequality is just the opposite one (see [11]). For them, $E_{0,1}>>mc^{2}$. We can now assess the total number *N* of baryons in the Universe via $N=K/mc^{2}$, with [13] $K=10^{53}\times c^{2}$ Joule. The result is $N∼6.25\times 10^{79}$.

In particular, $E_{0,1}∼10^{-21}$, and assuming that the major contribution of the baryonic pairs of gravitationally interacting baryons comes from their ground state, we can estimate that their contribution to dark matter is $E_{B}∼2\times 10^{77}$ eV, very small in comparison to the estimated value for dark matter of $K=2.86∼10^{84}$ eV [11]. In this last reference, it is seen that the gravitational interaction between axions does significantly contribute to the extant amount of dark matter.

**Figure 1:**
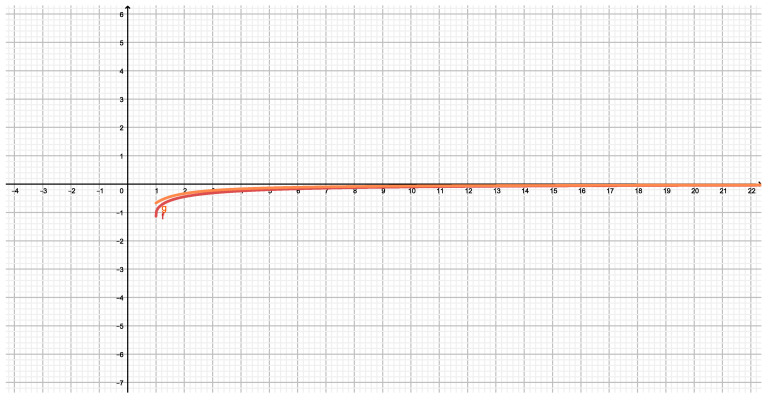
The red curve (f) represents to $E_{p}\left(x\right)/A$ given by (1), the orange curve (g) to V(x)/A given by (2) where $x=r/r_{2}$, for x>1 and $A=Gm^{2}/r_{2}$. We have selected x>1 to draw this graph.

Figure 1 displays the graphs of $E_{p}\left(x\right)/A$ in red (f) and V(x)/A in orange (g). $E_{p}\left(r\right)$ is given by (1) and V(r) by (2). The variable *x* is defined as $x=r/r_{2}$ with $r_{2}={\left(a/b\right)}^{1/3}$ and *A* is given by $A=Gm^{2}/r_{2}$. We have selected x>1 to draw the graph.

### 3.2. $V_{2}$’s Exact Treatment

We have

(17) $$U_{2}^{\prime \prime }\left(r\right)+\left[-\frac{l(l+1)}{r^{2}}+\frac{2m_{r}}{\hbar^{2}}\left[E-W\left(r\right)\right]\right]U_{2}\left(r\right)=0.$$

Four operating BC are operative here: $R_{l2}\left(r_{0}\right)=0,\;\;\;R_{l2}^{{}^{\prime }}\left(r_{0}\right)=0$, $R_{l2}\left(r_{1}\right)=0$, and $R_{l2}^{{}^{\prime }}\left(r_{1}\right)=0$, and we can only satisfy three of them. Accordingly, the only solution is $R_{l2}\left(r\right)=0$.

### 3.3. $V_{3}$’s Exact Treatment

We get

(18) $$U_{3}^{\prime \prime }\left(r\right)+\left[-\frac{l(l+1)}{r^{2}}+\frac{2m_{r}}{\hbar^{2}}\left(E+\frac{GmM}{r}\right)\right]U_{3}\left(r\right)=0.$$

It is of help here to remember that Whitaker’s function *W* solves the related differential equation

(19) $$W^{\prime \prime }+\left(-\frac{1}{4}+\frac{\lambda }{z}+\frac{\frac{1}{4}-\mu^{2}}{z^{2}}\right)W=0.$$

**Choose E<0**

Defining $\mu =l+\frac{1}{2}$, $\lambda =\frac{GmM}{\hbar }\sqrt{\frac{m_{r}}{2|E|}}$, it is clear that $s=\sqrt{\frac{8m_{r}\left|E\right|}{\hbar^{2}}r}$ for solving (18), one can write: *A* and *B* are arbitrary constants

(20) $$U_{3}\left(r\right)=AW_{\lambda ,\mu }\left(s\right)-BW_{-\lambda ,\mu }\left(-s\right),$$

where $W_{\lambda ,\mu }\left(z\right)$ is given by

(21) $$\begin{array}{c} W_{\lambda ,\mu }\left(z\right)=\frac{{\left(-1\right)}^{2\mu }z^{\mu +\frac{1}{2}}e^{-\frac{z}{2}}}{\Gamma \left(\frac{1}{2}-\mu -\lambda \right)\Gamma \left(\frac{1}{2}+\mu -\lambda \right)}\left\{\sum_{k=0}^{\infty }\frac{\Gamma \left(k+\mu -\lambda +\frac{1}{2}\right)}{k!(2\mu +k)!}\right.⊗\\ \left[\psi \left(k+1\right)+\psi \left(2\mu +k+1\right)-\psi \left(\mu +k-\lambda +\frac{1}{2}\right)-\ln z\right]+\\ \left.{\left(-z\right)}^{-2\mu }\sum_{k=0}^{2\mu -1}\frac{\Gamma \left(2\mu -k\right)\Gamma \left(k-\mu -\lambda +\frac{1}{2}\right)}{k!}{\left(-z\right)}^{k}\right\}. \end{array}$$

Here, 2μ+1 is a natural number. The last sum vanishes for μ=0. Accordingly,

(22) $$R_{l3}\left(r\right)=r^{-1}\left[AW_{\lambda ,\mu }\left(s\right)-BW_{-\lambda ,\mu }\left(-s\right)\right].$$

The operating BC here are $R_{l3}\left(r_{1}\right)=R_{l3}^{{}^{\prime }}\left(r_{1}\right)=0$. They can be translated into

(23) $$W_{\lambda ,\mu }^{{}^{\prime }}\left(s_{1}\right)+\frac{W_{\lambda ,\mu }\left(s_{1}\right)}{W_{\lambda ,\mu }\left(-s_{1}\right)}W_{-\lambda ,\mu }^{{}^{\prime }}\left(-s_{1}\right)=0.$$

Let $\sigma_{l,n}$ be the zeroes of such an equation. Then,

(24) $$s_{1}=\sigma_{l,n},$$

and the energy becomes quantized, as one should expect:

(25) $$E_{l,n}=-\frac{\hbar^{2}}{8m_{r}}\frac{\sigma_{l,n}^{2}}{r_{1}^{2}}.$$

**Choose E>0**

We have $\mu =l+\frac{1}{2}$, $\lambda =-i\frac{GmM}{\hbar }\sqrt{\frac{m_{r}}{2E}}$ $s=\sqrt{\frac{8m_{r}E}{\hbar^{2}}r}$. Now, the solution becomes

(26) $$U_{3}\left(r\right)=AW_{\lambda ,\mu }\left(-is\right)-BW_{-\lambda ,\mu }\left(is\right),$$

and then

(27) $$R_{l3}\left(r\right)=r^{-1}\left[AW_{\lambda ,\mu }\left(-is\right)-BW_{-\lambda ,\mu }\left(is\right)\right].$$

The operating BC are, once again, $R_{l3}\left(r_{1}\right)=R_{l3}^{{}^{\prime }}\left(r_{1}\right)=0$, that translate into

(28) $$W_{\lambda ,\mu }^{{}^{\prime }}\left(-is_{1}\right)+\frac{W_{\lambda ,\mu }\left(-is_{1}\right)}{W_{\lambda ,\mu }\left(is_{1}\right)}W_{-\lambda ,\mu }^{{}^{\prime }}\left(is_{1}\right)=0.$$

Denote by $\varsigma_{l,n}$ the zeroes of the above equation:

(29) $$s_{1}=\varsigma_{l,1}.$$

Energy becomes quantized again and the quantized eigenvalues become

(30) $$E_{l,n}=\frac{\hbar^{2}}{8m_{r}}\frac{\varsigma_{l,n}^{2}}{r_{1}^{2}}.$$

In the two cases considered in the present subsection, the separation between the quantum energy levels is of the order $10^{-17}$ Joules, meaning that we have a continuum energy, as should be expected.

### 3.4. $V_{4}$’s Exact Treatment

We have

(31) $$U_{4}^{\prime \prime }\left(r\right)+\left[-\frac{l(l+1)}{r^{2}}+\frac{2m_{r}}{\hbar^{2}}\left(E+\frac{2GmM}{3r}\right)\right]U_{4}\left(r\right)=0.$$

We again remember that Whitaker’s function *W* solves the related differential equation

(32) $$W^{\prime \prime }+\left(-\frac{1}{4}+\frac{\lambda }{z}+\frac{\frac{1}{4}-\mu^{2}}{z^{2}}\right)W=0.$$

**Choose E<0**

Defining $\mu =l+\frac{1}{2}$, $\lambda =\frac{2GmM}{3\hbar }\sqrt{\frac{m_{r}}{2|E|}}$, it is clear that $s=\sqrt{\frac{8m_{r}\left|E\right|}{\hbar^{2}}r}$ for solving (18), one can write: *A* and *B* are arbitrary constants.

(33) $$U_{4}\left(r\right)=AW_{\lambda ,\mu }\left(s\right)-BW_{-\lambda ,\mu }\left(-s\right),$$

and therefore

(34) $$R_{l4}\left(r\right)=r^{-1}\left[AW_{\lambda ,\mu }\left(s\right)-BW_{-\lambda ,\mu }\left(-s\right)\right].$$

$R_{l4}$ should verify $R_{l4}\left(r_{2}\right)=R_{l3}\left(r_{2}\right)$ and $R_{l4}^{{}^{\prime }}\left(r_{2}\right)=R_{l3}^{{}^{\prime }}\left(r_{2}\right)$.

Observe that the energy is not quantized in this case.

**Choose E>0**

We have $\mu =l+\frac{1}{2}$, $\lambda =-i\frac{2GmM}{3\hbar }\sqrt{\frac{m_{r}}{2E}}$ $s=\sqrt{\frac{8m_{r}E}{\hbar^{2}}r}$. Now, the solution becomes

(35) $$U_{4}\left(r\right)=AW_{\lambda ,\mu }\left(-is\right)-BW_{-\lambda ,\mu }\left(is\right),$$

and then

(36) $$R_{l4}\left(r\right)=r^{-1}\left[AW_{\lambda ,\mu }\left(-is\right)-BW_{-\lambda ,\mu }\left(is\right)\right].$$

The operating BCs are, once again, $R_{l4}\left(r_{2}\right)=R_{l3}\left(r_{2}\right)$ and $R_{l4}^{{}^{\prime }}\left(r_{2}\right)=R_{l3}^{{}^{\prime }}\left(r_{2}\right)$.

The energy is not quantized again.

## 4. Discussion

We have herein solved, for fermions, Schrödinger’s equation for gravity. The logic on which this paper was written can be summarized as follows.

- We began by adopting Verlinde’s stance that gravity emerges from an entropy *S* (entropic force);
- In [12], for a gas of free fermions, we obtained (1) *S*, (2) Verlinde’s entropic force $F_{e}$, and from it (3) gravity’s potential V(r). We also found in [12] that V(r) differs from Newton’s form at extremely short and extremely large distances.

The above potential V(r) was approximated in a suitable manner so as to be in a position to obtain analytical solutions to the pertinent SE for the potential V(r).

The novel results of our treatment emerge at short distances (the $V_{1}$ component of V(r)). The ensuing low-lying SE-quantum states yield energy eigenvalues (most importantly, the ground state $E_{0,1}$, not accounted for before). They produce, via Einstein’s relation energy $=mc^{2}$, a quantity of matter that we might identify as dark. However, this Schrödinger-baryonic dark mass is insignificant. Therefore, baryons do not contribute to dark matter in this from of gravity, which, we believe, constitutes an important result, because bosons do contribute [11].

## References

[1] Verlinde E. (2011). On the Origin of Gravity and the Laws of Newton. arXiv.

[2] Plastino A., Rocca M.C. (2018). On the entropic derivation of the *r*^−2^ Newtonian gravity force. Physica A.

[3] Overbye D. (2010). A Scientist Takes On Gravity. The New York Times.

[4] Calmthout M. (2010). Gravity’s origin falling into place. New Sci..

[5] Makela J. (2010). Notes Concerning ”On the Origin of Gravity and the Laws of Newton” by E. Verlinde. arXiv.

[6] Lee J. (2010). Comments on Verlinde’s entropic gravity. arXiv.

[7] Kiselev V.V., Timofeev S.A. (2010). The Surface Density of Holographic Entropy. Mod. Phys. Lett. A.

[8] Padmanabhan T. (2009). Statistical mechanics of gravitating systems: An Overview. arXiv.

[9] Guseo R. (2016). Diffusion of innovations dynamics, biological growth and catenary function. Physica A.

[10] Verlinde E. (2011). The Hidden Phase Space of our Universe. arXiv.

[11] Plastino A., Rocca M.C. (2019). Statistical Mechanics’ Schrödringer Treatment of Emergent Entropic Forces. https://www.researchgate.net/publication/331977251_Statistical_Mechanics’_Schrodringer_treatment_of_emergent_entropic_forces.

[12] Plastino A., Rocca M.C. (2019). Spatial cut-offs, Fermion statistics, and Verlinde’s conjecture. Physica A.

[13] Brooks J. (2014). Galaxies and Cosmology.

[14] Smullin S.J., Geraci A.A., Weld D.M., Kapitulnik A. Testing Gravity at Short Distances. Proceedings of the SLAC Summer Institute on Particle Physics (SSI04).

[15] Gradshteyn I.S., Ryzhik I.M. (1980). Table of Integrals, Series and Products.

